# Time Series Complexities and Their Relationship to Forecasting Performance

**DOI:** 10.3390/e22010089

**Published:** 2020-01-10

**Authors:** Mirna Ponce-Flores, Juan Frausto-Solís, Guillermo Santamaría-Bonfil, Joaquín Pérez-Ortega, Juan J. González-Barbosa

**Affiliations:** 1 Graduate Program Division, Tecnológico Nacional de México/Instituto Tecnológico de Ciudad Madero, Cd. Madero 89440, Mexico; jjgonzalezbarbosa@gmail.com; 2 Information Technologies Department, Consejo Nacional de Ciencia y Tecnología—Instituto Nacional de Electricidad y Energías Limpias, Cuernavaca 62490, Mexico; 3 Computing Department, Tecnológico Nacional de México/Centro Nacional de Investigación y Desarrollo Tecnológico, Cuernavaca 62490, Mexico; jpo.cenidet@gmail.com

**Keywords:** classical forecasting methods, complexity, entropy, error measures, symbolic analysis, M4 competition

## Abstract

Entropy is a key concept in the characterization of uncertainty for any given signal, and its extensions such as Spectral Entropy and Permutation Entropy. They have been used to measure the complexity of time series. However, these measures are subject to the discretization employed to study the states of the system, and identifying the relationship between complexity measures and the expected performance of the four selected forecasting methods that participate in the M4 Competition. This relationship allows the decision, in advance, of which algorithm is adequate. Therefore, in this paper, we found the relationships between entropy-based complexity framework and the forecasting error of four selected methods (Smyl, Theta, ARIMA, and ETS). Moreover, we present a framework extension based on the Emergence, Self-Organization, and Complexity paradigm. The experimentation with both synthetic and M4 Competition time series show that the feature space induced by complexities, visually constrains the forecasting method performance to specific regions; where the logarithm of its metric error is poorer, the Complexity based on the emergence and self-organization is maximal.

## 1. Introduction

Presently, time series forecasting is applied to many areas such as weather, finance, ecology, health, electrochemical reactions, computer networks, and so on [1]. Among the most popular and effective methods stand the classical time series models such as the Simple Exponential Smoothing (SES) and the Autoregressive Integrated Moving Average (ARIMA). Also, forecasting methods of machine learning such as Neural Networks have gained popularity after the results of the Smyl winning method of the M4 Competition, and the benchmark forecasting methods of Theta, ARIMA, and ETS [2]. In the forecasting area, researchers agree that it is too difficult to identify a suitable forecasting method for a particular time series beforehand, even knowing its specific statistical characteristics [3]. For instance, time series (TS) complexity [3] is a widely debated measure, which it is supposed to quantify the *intricacy* of the time series, allowing choice of the forecasting methods to be applied. Shannon’s entropy has been used to measure the complexity of discrete systems [4]. Although the entropy formula was conceived in the thermodynamic area, the entropy concept has spread to different disciplines adapting its meaning in regard to the applied area and making tools for many applications [5,6,7]. For example, in [8] a package with functions to measure emergence, self-organization, and complexity applied to discrete and continuous data is presented as a framework; the present study is based on them. However, to the best of the authors’ knowledge, these formulae have not been applied to assess the *forecastability* of time series. Furthermore, this framework is extended with other measures. We present four complexity measures based on entropy and a methodology for determining the relationships between these complexity measures and the forecasting error of the Smyl [9], Theta [10], ARIMA [11,12], and ETS [13] methods; all of them were participants of the M4 Competition [14]. This study was made for a dataset with some synthetic time series [15] and more than 20,000 time series taken from M4 Competition [16], which is a reference point used by many researchers. We obtain the prediction error with the forecasting values of each one of the four selected methods, and we determine four complexity measures based on the relationship between Entropy and Mean Absolute Scaled Error (MASE) error [17], but for functionality we use the logarithm values of MASE error log(MASE). We present a complexity log(MASE) analysis, and we apply a visualization method [18] for the time series of the dataset. Finally, the experimentation shows that the permutation and 2-regimen complexities are the measures that identify patterns of the distribution of TS on the two-dimensional space; also we found a relationship between the permutation complexity and the log(MASE) values and finally we make a comparison between the four forecasting methods reinforcing the known *No-free lunch* theorem.

This paper is organized as follows. Section 2 presents the materials used in this research; Section 3 describes the methods, parameters settings, methodology and the dataset used in the experimentation. In Section 4, we provide the results of the experimentation. Finally, Section 5 presents the conclusion for this work.

## 2. Materials

The complete dataset of the time series used in this paper is divided into two subsets: Synthetic and M4 Competition. Each of these is described in the following subsections.

### 2.1. Synthetic Time Series

Three generating mechanisms were considered for the construction the of synthetic TS: (a) sine waves; (b) logistic map; and (c) a time series tool, namely *GRATIS* [15]. It is worth noting that in order to generate time series belonging to the same mechanism type, either the parameters of the generating function are modified or noise is introduced at a certain specific Signal-to-Noise Ratio (SNR). The synthetic TS considered are *Sine Wave corrupted by uniform noise*, *Sine Wave corrupted by Gaussian noise*, *1-D logistic Map*, and the *GRATIS tool*.

#### 2.1.1. Sine Waves TS

A stationary and seasonal TS is generated using a sine wave of the form:

(1) $$x_{t}=\alpha \cdot sin\left(\omega t\right),$$

where $x_{t}$ is the observation at time *t*, α corresponds to the wave amplitude parameter, and ω to the wave frequency. A family of time series is spawned from Equation (1) by corrupting the wave at specific SNRs. In the case of the latter, the contaminated series is defined as

X=f(x)+k·ϵ,

where f(x) is the sine wave, *k* is an increasing constant, and ϵ∈P(X) is a shock which follows a uniform or Gaussian distribution. In these cases, the SNR is determined by

$$SNR=\frac{Var(f(x))}{Var(\epsilon )},$$

where larger values of SNR imply that it is easier to detect the signal, and smaller values otherwise.

#### 2.1.2. Logistic Map TS

The logistic Map is a 1-dimensional chaotic dynamic system which is commonly employed as benchmark to study tools and methods used to characterize chaotic dynamics [19,20,21]. The logistic map function is defined as

(2) $$x_{t+1}=r\cdot x_{t}\left(1-x_{t}\right),$$

where $x_{0}$ is a random number within $0<x_{t}<1$, and *r* is a constant. In fact, this last parameter is the one that defines the behavior of Equation (2). More precisely, when r<1 the system always collapses to zero, for 1≤r≤3 the system tends to a single value, for 3<r<3.6 the system is fixed to period-doubling points, and from r∼3.6 the system exhibits a chaotic behavior.

#### 2.1.3. GRATIS TS

The last subset of time series was generated using the GRATIS tool [15] that is based on Gaussian Mixture Autoregressive (MAR) models. These models contain multiple stationary or non-stationary autoregressive components, non-linearity, non-Gaussianity, and heteroscedasticity. To tune the parameters for MAR models, the GRATIS’ authors use a Genetic Algorithm when the distance between the target feature vector and the feature vector is close to zero. This tool generates time series with diverse parameters such as length, frequency, and behavior features.

### 2.2. M4 Competition TS

The complete set is composed of 100,000 real-life series divided into sets named “periods” (Yearly, Quarterly, Monthly, Weekly, Daily, and Hourly) and subdivided into subsets or types (Demographics, Finance, Industry, Macro, Micro, and Other). Our criterion for selecting time series was: (1) To choose time series with a minimum of two hundred and 50 observations; (2) The frequency group should have more than one set type. Consequently, TS from the Hourly group were not selected since it only contains time series from the type *Other*. The complete dataset is shown in Table 1, which has two final columns named size and percentage (%); the former refers to the number of time series selected from each frequency group, and the latter is the correspondent percentage of selected time series concerning that group. According to the last criterion, in our dataset, we consider only the subsets Yearly, Quarterly, Monthly, Weekly, and Daily; the total number of the TS in our dataset is 22,610.

## 3. Methods

To analyze the relationship between the prediction errors of classical forecasting methods such as ARIMA, we build a feature space [18] based on Shannon entropy (*H*) features that presumably can be used to identify those TS instances where ARIMA forecasting errors are expected to be higher or lower, accordingly. These features are based on four entropy-based complexity measures, namely the frequentist binning approach ($H_{dist}$) [22]; 2-Regimes ($H_{2reg}$) [19] and Permutation ($H_{perm}$) entropy [23]. These three built upon the notions of symbolic dynamics, and the Spectral entropy ($H_{spct}$) [24] based on the analysis of the spectrum of a time series. The main difference between these measures is the *discretization* or *symbolization* followed to describe the states of dynamical systems, which has a deep impact on the quantification of entropy [25]. For instance, consider a TS with hundreds of points coming from a sine wave; with the frequentist binning approach, we will be studying a system whose probability distribution follows an arc-sine distribution, whereas if we represent it by symbols that correspond to 1-period waves, we will be studying a system which follows a Dirac delta probability distribution.

On the other hand, $H_{dist}$ has been used to study a dynamical system in terms of the rate of Emergence (*E*) of new states or information, the rate of Self-organization (*S*) displayed as discernible patterns, and the interplay between these two called Complexity (*C*), hereafter ESC for short [8]. In particular, systems with higher *C* concentrates its dynamics into a few highly probable states with many less frequent states [8]. In this work, the ESC framework is extended to study the interplay between *E* and *S* for $H_{spct}$, $H_{2reg}$, and $H_{perm}$.

Therefore, first, the Shannon-based complexity measures and TS symbolization for each is presented; second, the ESC framework is introduced along with the Complexity Feature Space; third, the forecasting methods are briefly defined; finally, the proposed analysis methodology is detailed.

### 3.1. A Background on Entropies

Entropy is a term with many meanings, but in the information theory area it usually refers to the average ratio of uncertainty a process produces, which is measured by the well-known discrete Shannon Entropy equation [4,26].

(3) $$H=\sum_{i=1}^{n}p_{i}log_{a}p_{i},$$

where *H* stands for Shannon Entropy, *n* is the total number of TS observations, *a* is the logarithm base, and $p_{i}$ is the probability for each symbol of the TS alphabet. It is worth noting that *information* may refer to the capacity of a channel for transmitting messages, the consequence of a message, the semantic meaning conveyed by it, and so on, all regardless of its specific meaning [27]. Entropy-based measures are the first option when the task at hand is the quantification of the *complexity* of a time series [28]. However, what does *complexity* stand for?

Complexity science is a multidisciplinary field in charge of studying dynamical systems composed of several parts, whose behavior is nonlinear, and that cannot be studied neither by the laws of linear thermodynamics nor by modelling parts in isolation [29,30]. A key aspect of these systems is that individual parts’ interactions will heavily determine the future states of the overall system, and shall induce spatial, functional, or temporal structures all alone (i.e., self-organizing) [27]. Similarly, these systems are considered *open* since they exchange matter, energy, and information with their environments [27,30]. These are observed in a multitude of disciplines such as biology, ecology, economy, linguistics, and so on; it is common to study their dynamical behavior through the observation of one or more of its variables in the form of TS [31].

There are several measures of *complexity*, but at its core remains the notion of information altogether to some form of Shannon entropy formulation [8]. These two notions spawn a myriad of complexity measures; among them stand out $H_{perm}$, the Kolmogorov–Sinai (KS) complexity, $H_{spct}$, $H_{2reg}$, Transfer entropy, LMC complexity, ϵ-complexity, ESC, and so on [8,27,31,32,33]. The diversity of such measurements is given by the inexorable subjectivity of what shall be considered *complex*, which is translated into a specific *quantization* of a TS regarding an observer point of view [25,27].

**Figure 1:**
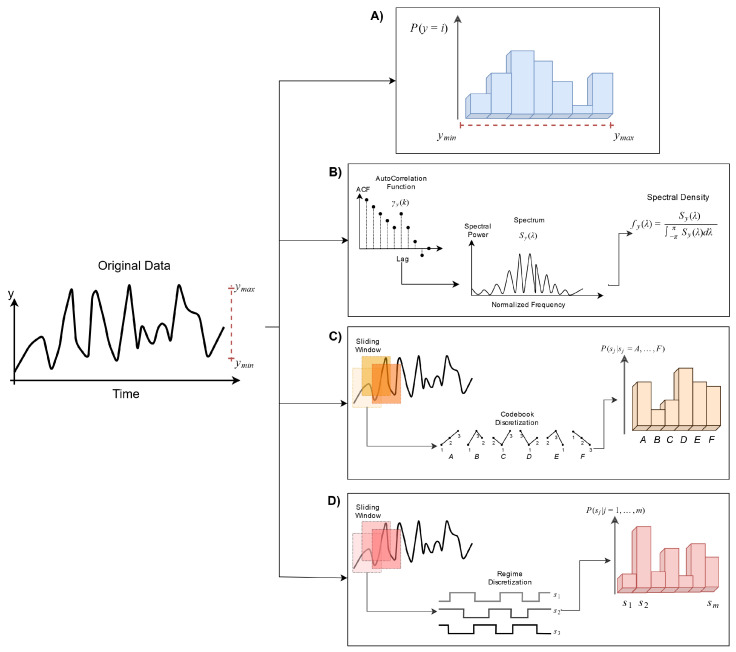
Four possible characterizations of the states of a dynamical system. On (**A**) the frequentist binning approach; on (**B**) the spectral probability density of the TS is estimated by the classical Fourier transform of the Auto Correlation Function (ACF); on (**C**,**D**) symbolic transformations define the alphabet by ordinal rank patterns and sequences of the first derivative sign, respectively.

In this work, *quantization* stands for the procedure to estimate the discrete probability distribution from a TS; in other words, how we describe the states of the system. In the classical *H*, continuous measurements are typically transformed into discrete states by binning measurements into non-overlapping ranges. To emphasize this form of entropy estimation *per se*, it will be referred as $H_{dist}$ and will reserve *H* for the concept. However, there are other ways in which we can discretize a time series into a probability distribution, which needs to be hand in hand with the properties of the time series that are analyzed. In Figure 1, a cartoon of the four symbolizations used in this work is shown.

#### 3.1.1. Spectral Entropy

Power Spectral Density (PSD) estimation is commonly used in signal-processing literature. By transforming a given time series $x_{t}$ from the time domain to the frequency domain using the discrete Fourier transform, the latter provides information about the power of each frequency component. These frequencies describe a spectral probability distribution which can be used to assess the uncertainty about a future prediction, namely spectral entropy $H_{spct}$ (a cartoon of this process is shown in Figure 1B). To calculate this from a TS (assumed to be stationary), we first require its Autocovariance Function (ACVF). This is defined as

(4) $$\gamma_{x}\left(k\right)=E\left[\left(x_{t}-\mu_{x}\right)\left(x_{t-k}-\mu_{x}\right)\right],\;k\in Z,$$

where $\mu_{x}$ is the TS mean value and *k* corresponds to the lag. With the ACVF, the spectrum of the TS is obtained through the Fourier transform such as

(5) $$S_{x}\left(\lambda \right)=\frac{1}{2\pi }\sum_{j=-\infty }^{\infty }\gamma_{x}\left(j\right)e^{ij\lambda },\;\lambda \in \left[-\pi ,\pi \right],$$

where $i=\sqrt{-1}$ and $S_{x}:\left[-\pi ,\pi \right]\to R^{+}$. To understand the implications of Equation (5) consider a white noise TS ω. In such case, $\gamma_{\omega }\left(k\right)=0$ for k≠0, thus, the spectrum is constant for all λ∈[−π,π] [24]. Then, if we define $\sigma_{x}^{2}=\int_{-\pi }^{\pi }S_{x}\left(\lambda \right)$ for k=0, the spectral density of $x_{t}$ is

(6) $$f_{x}\left(\lambda \right)=\frac{S_{x}\left(\lambda \right)}{\sigma_{x}^{2}}=\frac{1}{2\pi }\sum_{j=-\infty }^{\infty }\rho_{x}\left(j\right)e^{ij\lambda },$$

where $\rho \left(k\right)=\frac{\gamma (k)}{\gamma (0)}$ corresponds to the Autocorrelation Function (ACF). The $f_{x}\left(\lambda \right)$ can be used as a probability density function of a random variable such that it is ascribed to the unit circle. For instance, in the case of ω, $f_{x}\left(\omega \right)=\frac{1}{2\pi }$, which corresponds to the spectral density of the uniform distribution [24].

Using Equation (6), the spectral entropy $H_{spct}$ is defined as

(7) $$H_{spct}=\int_{-\pi }^{\pi }f_{x}\left(\lambda \right)log_{a}f_{x}\left(\lambda \right)d\lambda .$$

If the value obtained by Equation (7) is relatively small, $x_{t}$ is more *forecastable* since it contains more signal than noise, whereas a larger value stands otherwise [18]. The previous analysis can be simplified by normalizing $H_{spct}$ within $0\leq H_{spct}\leq 1$ by dividing it by the uniform distribution entropy i.e., $log_{a}\left(2\pi \right)$ (which has the maximum entropy for a finite support). In this sense, the uncertainty about a required prediction $x_{t+h}$ is given by the characteristics of the process itself [24].

#### 3.1.2. Permutation Entropy

Permutation Entropy ($H_{perm}$) was conceived by Bandt and Pompe as an entropy-based measure for measuring the *complexity* of a TS [23]. $H_{perm}$ is based on the concepts of *H* and Symbolic Dynamics (SD). In contrast to $H_{dist}$ and $H_{spct}$, $H_{perm}$ does not ignore temporal information.

The SD carried to obtain $H_{perm}$ consists of transforming TS data into a sequence of discrete symbols, i.e., length-L Ordinal Patterns (OP). These are produced by encoding consecutive observations contained in a sliding window of size L,L≥2, into *permutations* determined by observations rank order in each window [20,34]. However, to determine the L-window, a Phase Space Reconstruction (PSR) needs to be carried out [28,35]. Such reconstruction employs two parameters—the embedding dimension $d_{e}$ and the time delay τ. Formally, given a TS of the form $X=x_{1},x_{2},\ldots ,x_{t}|x_{i}\in R$, a point mapped to the reconstructed $d_{e}$-dimensional space is of the form $\vec{x_{j}}=\left\{x_{t},x_{t-\tau },\ldots ,x_{(d_{e}-1)\tau }\right\}|\vec{x_{j}}\in X_{R}$, thus, $L=(d_{e}-1)\tau$.

Once TS is mapped into this space, portrait permutations are obtained. A permutation $\pi_{j}\in \Pi$ is given by the permutation of indices (from 0 to $d_{e}-1$), which puts the $d_{e}$ values of a given $\vec{x_{j}}$ into ascending sorted order. Notice that there are $d_{e}!$ different permutations. Afterwards, the Permutation Distribution (PD), also known as *codebook*, is calculated by counting the relative frequency of each symbol. Analyzing the behavior of a TS by its PD has several advantages: it is invariant to monotonic transformations of the underlying TS, requires low computational effort, is robust to noise, and does not require knowledge of the data range beforehand [20,35].

Once the PD is estimated, $H_{perm}$ is obtained such that

(8) $$H_{perm}=-\sum_{\pi_{j}\in \Pi }\pi_{j}log_{a}\pi_{j},$$

where Π is the set of all different $d_{e}$ permutations. A cartoon of this process is shown in Figure 1C.

For convenience, $H_{perm}$ can be normalized by dividing it by $log_{a}\left(d_{e}!\right)$ to constrain it to $0\leq H_{perm}\leq 1$. In this sense, a lower value of the normalized $H_{perm}$ corresponds to more regular and deterministic process, whereas a value closer to 1 is observed in more random and noisier TS. Notice that $H_{perm}$ is closely related to the Kolmogorov–Sinani (KS) entropy and equal to it when the TS is stationary. However, in contrast to KS entropy, $H_{perm}$ it is computationally feasible to calculate $H_{perm}$ for long L-windows [21,28,34].

#### 3.1.3. 2-Regimes Entropy

Under SD, a *regime* stands for a qualitative behavior defined as a growth model or dynamical rule with its own state space that allows the existence of multiple attractors in equilibrium or not, at the same time [19]. This *symbolization* (transforming TS values into symbols) allows study, for instance, of structural changes in a TS such as sudden changes in trend, such as changes in the governing rules of a dynamical system (e.g., switching between trends). An adequate symbolization allows the highlighting of temporal patterns, to improve the signal-to-noise ratio, improve computation efficacy and efficiency, to mention a few [36]. To understand this, consider a time series of the form $X=x_{1},x_{2},\ldots ,x_{t}|x_{i}\in R$. Typically, the *symbolization* of a TS is carried out by dividing R into q≥1|q∈{1,2,…} non-overlapping bins. Such bins represent the states of the system; hence, each $x_{i}$ is mapped to its corresponding partition, mapping from a sequence of points *X* into a sequence of symbols $Z=z_{1},z_{2},\ldots ,z_{t}$. In the simplest case when q=2, the original TS is mapped into a sequence of two symbols (i.e., 0 or 1) using a threshold that partitions R into two intervals, namely *2-regimes* symbolization. This representation is useful to study trends of growth (expansion) or fall (contraction) in a TS, for instance the *bear* and *bull* regimes in an economic market. In this work, 2-regime symbolization is carried out by employing the sign of the first difference such that

(9) $$z_{t}=\left\{\begin{array}{ll} 1 & 1\cdot sgn(x_{t}-x_{t-1})>0,\\ 0 & \mathrm{otherwise}, \end{array}\right.$$

where sgn stands for the sign function. An example of this codification is presented in Table 2, where the first row displays the original observations, and the second the corresponding 2-regimes codification. Notice that due to Equation (9), the length of the codified TS is n-1.

Once the TS is symbolized into *Z*, Equation (3) can be employed to calculate a two-regime entropy ($H_{2reg}$). It is worth mentioning that $H_{2reg}$ can be considered to be a special case of $H_{perm}$, i.e., during the symbolization step of a TS, a permutation with $d_{e}!=2$ will produce equivalent symbols to those obtained by Equation (9). However, by considering sequences of contiguous symbolized observations $z_{t},z_{t+1},\ldots ,z_{t+d}$, an alphabet of size $2^{d}$ can be explored, allowing study of richer 2-regimes alphabets. In this work, the alphabet size is set to $2^{d}=256,\;d=8$. Finally, notice that $H_{2reg}$ can be normalized by $log_{a}\left(2^{d}\right)$ to constrain it within $0\leq H_{2reg}\leq 1$. In this sense, a lower value is obtained when there is a predominant regime (e.g., trend or drift); a value closer to one stands for a more random and noisier TS.

### 3.2. ESC and the Complexity Feature Space

Regarding the *forecastability* (i.e., determining a system future states) of a TS $\Omega (x_{t})$, some complexity measures such as $H_{spct}$, defines it as the complement of the average uncertainty of the process (given by its spectral density) such that $\Omega (x_{t})=1-H*$, where H∗ corresponds to the normalized version of $H_{spct}$ [24]. On the other hand, others indicate that the *forecastability* shall be in terms of existing and new patterns. Thus, complexity may be defined as the relationship between stability and instability [30], Information and Disequilibrium [32], redundancy and new information [21], or Emergence and Self-organization [29]. In particular, it has been established that among the basic properties of complex systems stand the emergence, self-organization, and complexity [27]. Therefore, here we decided to extend the ESC paradigm using different entropy functions, namely $H_{spct}$, $H_{perm}$, and $H_{2reg}$. In this sense, it is possible to measure (1) the average uncertainty given by a probability distribution considering multiple quantizations, (2) estimate their compliments associated with the *forecastability*, and (3) analyze the interplay between these two.

Formally, the Emergence (E), Self-organization (S), and Complexity (C) for a TS, irrespectively of the entropy of choice, is given by the following

(10) $$E=-K\cdot H^{p}\left(x_{t}\right)$$

(11) S=1−E,

(12) C=4·E·S,

where $H^{p}\left(x_{t}\right)$ is the normalized version of $H_{dist}$, $H_{spct}$, $H_{perm}$, or $H_{2reg}$, such that 0≤E,S,C≤1. This normalization is carried by the constant $K=\frac{1}{log_{a}\left(U_{b}\right)}$, which corresponds to the entropy of uniform distribution with an alphabet of size b. It is worth mentioning that when required, *E*, *S*, and *C* for a particular entropy mentioned above will be referred to with the entropy ID underscored. For instance, if we refer to (E,S,C) tuple for $H_{spct}$, these may be referred as $\left(E_{spct},S_{spct},C_{spct}\right)$, respectively.

The feature space conformed by these 12 measures is called the Complexity Feature Space (CFS). Hence, any TS is now mapped to a 12-D space and given the aforementioned definitions of complexity, and is expected that in the CFS it will be grouped into a specific region in accordance with its *forecastability*. Notice that such a region will depend, in part, of the model used to forecast [21] as well as the forecasting horizon. However, to obtain any information from the CFS regarding the relationship between forecastability and complexity, it is a necessary tool for its analysis. For that matter, visual tools based on a dimensional reduction technique such as Principal Components Analysis (PCA) can be employed [18]. In this sense, any TS from the CFS are now displayed as 2-D points whose dimensions correspond to two principal component axes. Although PCA leads to a loss of information due to its linear nature, the topological distribution of points is mostly preserved [18]. Finally, this feature space can be improved by considering other entropy-based complexity measures such as Transfer Entropy [37] or Tsallis Entropy [38] or different characteristics such as the trend, frequency, or seasonality [2,18,39,40].

### 3.3. Forecasting Methods: Smyl, Theta, ARIMA and ETS

On M4 Competition, 61 forecasting methods participated, the sharing dataset contains in addition the forecast values for the better 25 methods. We select four of them, considering the Smyl winning method and three classical benchmark methods; each of them is described in the next paragraph, and ordered according to the final position in the competition.

- Smyl: This is a hybrid method that combines exponential smoothing (ES) with recurrent neural network (RNN); this method is called ES-RNN [9] and is the winning method for M4 Competition.
- Theta: was one of winning methods on M3, the previous competition, and in the past was indicated to be a variant of the classical exponential smoothing method [10].
- ARIMA (Autoregressive Integrated Moving Average): It is one of the most widely used by the Box & Jenkings methodology [41], mainly applied for nonlinear patterns in TS.
- ETS (exponential smoothing state space [13]): This method is especially used in forecasting for TS that presents trends and seasonality.

The ARIMA method is used to forecast all complete datasets, including synthetic and M4 Competition TS, and the other three methods are used only for M4 Competition TS.

### 3.4. Analyzing the Forecasting Performance in the CFS

**Figure 2:**
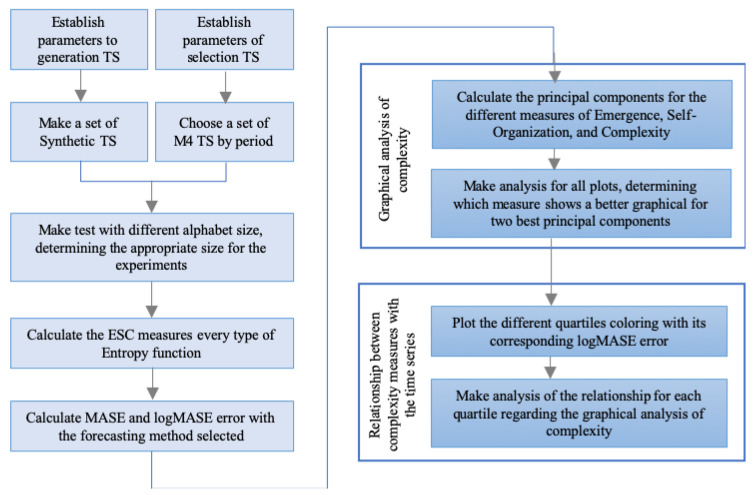
Proposed method.

A global view of the executed steps to build the CFS for analyzing the relationship between TS forecastability and complexities is presented in Figure 2.

The first step consists of gathering TS. In our case, we tested the CFS using two types of data sets: synthetic, and M4 Competition TS. Afterward, parameters of TS complexity measures, such the alphabet size, is determined. The third step consists of the calculation of the ESC for every type of Entropy function. Recall that these produce a total of 12 measures per TS. The latter is repeated for each TS that belongs to the set (either Synthetic or selected M4 Competition TS). The fourth step is to make the forecast for each TS in accordance with its corresponding forecasting horizon, and measure its error using a performance measure. In the fifth step the ESC measures of the dataset along with PCA are used to build the CFS to visually display TS in 2-D. Finally, in the last step the performance metric is displayed in the 2-D CFS to assess its relationship with the complexity measures. To enhance this step, the relationship between forecastability and complexity is assessed by plotting quartiles of the performance metric.

#### Parameters Settings

So far, we have neglected some details regarding the parameters to build the CFS to analyze forecasting performance. First, we detail the parameters used to generate synthetic TS data. Then, entropy-based parameters and the performance metric are presented.

All synthetic TS have $10^{4}$ observations, all sine waves have an amplitude of α=1, and frequency of ω=2. SNRs for both TS corrupted by uniform and Gaussian noise are $SNR=10^{-3},10^{-2},10^{-1},10^{-0.9},10^{-0.5},10^{-0.1},1,10$. In particular, for the Gaussian noise, we used a standard deviation of $\sigma^{2}=1$. Thus, for each sine wave, we generated 8 TS, giving a total of 16 sine waves. Regarding the logistic map, we employed an r∈[3.1,4] such that Δr=0.005 with a starting point of $x_{0}=0.1$ which produces 181 TS. The last subset of synthetic TS is 16 TS generated with the tool GRATIS described in a previous section. We choose parameters like length equal to 600, spectral entropy between 0.25 and 1.00 rank, and Monthly frequency. In total, the Synthetic dataset is composed of 215 TS. On the other hand, we selected a subset of the M4 Competition data composed of 22,610 TS. Regarding the forecasting horizon, for the M4 Competition TS, the dataset contains for each TS a training and test observation part, and a defined horizon as well, thus, the Yearly period has an horizon of 6, Quarterly 8, Monthly 18, Weekly 13, and Daily 14, considering that the synthetic TS generated corresponds to Monthly period, following the same scheme of M4 Competition, the horizon selected was of 18.

Regarding the entropy-based complexities, there are some parameters to be established beforehand. In the case of $H_{spct}$ we employed the implementation of the package *forecast* [42], which is an already normalized version of entropy i.e., $0\leq H_{spct}\leq 1$. In contrast, $H_{dist}$, $H_{2reg}$, and $H_{perm}$ require selection of an alphabet size. It is worth mentioning that the selection of the alphabet length was rather arbitrary, and perhaps it is a parameter for tuning or taking advantage. In the case of $H_{dist}$ and $H_{2reg}$ we used an alphabet size of d=8; thus, the alphabet size was $2^{d}=256$. The case of $H_{perm}$ is special, since it will depend on the time series and the reconstructed phase space. For such purpose, the Mutual Information method and the Approximate Nearest Neighbor method are employed to estimate τ and $d_{e}$, respectively, in accordance with Cao’s practical method [43]. In particular, for the logistic map $\tau =2,d_{e}=8$ in accordance to [28], in this case the alphabet size is 8!=40,320 permutations (however only those $P(\pi_{j})>0$ are considered). To estimate the PD required by $H_{perm}$ the package *pdc* is employed [35]. The $H_{2reg}$ and ESC measures from the entropy-based complexities were calculated using a self-implementation in R based on [8]. To estimate the error of forecasting methods, we use forecast values of 4 methods provided in *M4comp2018* package. The MASE is calculated using the *forecast* package. Furthermore, during the experimentation we noticed a logarithm relationship between the MASE and some ESC values, thus, MASE values are scaled $log_{10}$ to highlight this relationship.

## 4. Results

To understand the relationship between the CFS and prediction performance for a given model, experiments were carried out using two different datasets: synthetic and M4 TS. For the first case, the purpose is to understand the relationship between ESC complexities against data whose underlying mechanisms can be controlled. In the second case, the value of the CFS in a real-world setting is explored to obtain a better idea of its potential in identifying regions (perhaps groups) of forecastability.

### 4.1. Complexities and Forecastability of the Synthetic TS

This section is divided into two subsections that are described below. The forecastability is analyzed only with the ARIMA forecasting method, which was executed from the *forecast* package. However, its parameters *p, r, and q* are tuned by following the procedure in [44]. This method is executed with *ARIMA* function with different combinations of p∈[0,10],d∈[1,3],q∈[0,10], and selecting those that obtained the smallest Akaike Information Criterion (AIC) value, all of these trying to obtain the better forecast for each TS that belongs to the subset of synthetic TS.

#### 4.1.1. The Logistic Map

**Figure 3:**
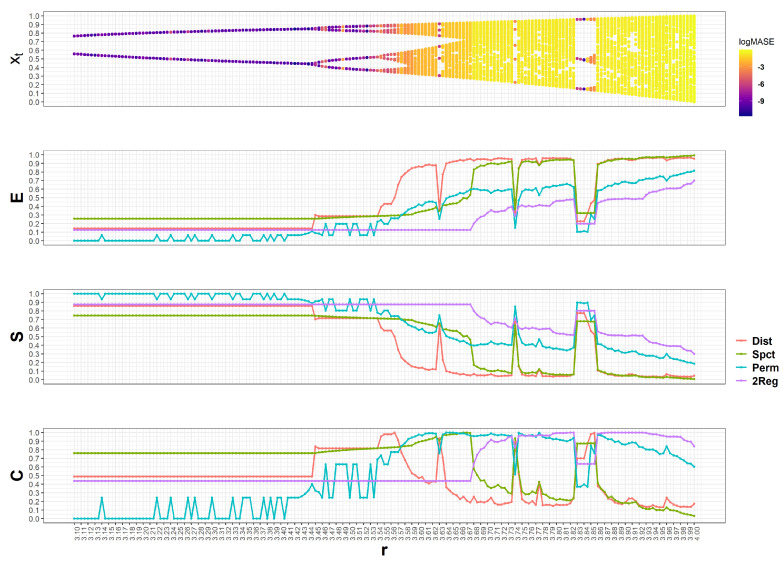
The Logistic Map and its ESC (Emergence, Self-Organization, and Complexity). The top plot shows the bifurcation diagram, whereas below the corresponding ESC for different entropy measures is showed.

To start the discussion of synthetic TS results, the logistic map is analyzed. This is a common benchmark used for the elucidation of the relationship between entropy-based complexities and forecastability [20,21,28]. Hence, the well-known Feigenbaum diagram along with its corresponding ESC measurements (from top to bottom) obtained for the logistic map are shown in Figure 3. Recall that the Feigenbaum diagram is a visual summary of the values ($x_{t}$) visited by a system as a function of a bifurcation parameter. Thus, in this case, as the parameter *r* grows the logistic map transitions from permanent oscillations between fixed-point pairs to the chaotic regime. Colors in the Feigenbaum diagram correspond to the log(MASE) obtained by an ARIMA model: lower errors are shown in dark blue whereas those with higher values are displayed in bright yellow. Hence, as the logistic map dynamics becomes more chaotic, the TS become less forecastable by the ARIMA model.

For the ESC plots, colors correspond to different entropy-based complexities: red for $H_{dist}$, green for $H_{spct}$, blue for $H_{perm}$, and purple for $H_{2reg}$. Observe that all entropy-based complexities are constant when oscillating between two values (r≤3.44), except for $H_{perm}$. In this case, ESC values using a binary alphabet shall be (E=1,S=0,C=0) for $H_{dist}$, $H_{2reg}$, and $H_{perm}$, however, by forcing an arbitrary large alphabet size, the self-organization is revealed. In fact, for $H_{perm}$ and r≤3.44, (E=0,S=1,C=0) for most cases consequence of a Dirac delta PD, with the exception of some spikes in which new ordinal patterns emerge. Observe that several of these spikes have worse log(MASE) than those obtained by contiguous *r* values. $H_{dist}$ grows immediately after r=3.44 due to doubling of the limit cycle, but remains steady until r=3.54, this contrasts to $H_{2reg}$ which does not grow, and $H_{spct}$ and $H_{perm}$ which increases slower. In fact, $H_{dist}$ seems to be a more sensitive measure to the alphabet size and not necessarily TS intricacy, since its *E* becomes very high between [3.54,3.63] in comparison to the rest of the complexities. Eventually, $H_{spct}$ and $H_{dist}$ concur in that the emergence of new states (E∼1) (or complementary, the reduction in self-organization S∼0) is similar to a random process. Conversely, $H_{2reg}$ and $H_{perm}$ increase slower as *r* grows, although the former does not change until r∼3.68 indicating that regimes displayed by the logistic map for r≤3.68 are constant.

The interplay between new states and the self-organization of the system, displayed by *C* is very interesting. For $H_{dist}$, when the logistic map has 2 fixed points (r≤3.44) a C=0.5 is obtained, when the period doubles it increases to C∼0.8, and it shows maximal complexity (C∼1) at points between double-periods and chaotic regimes (*at the edge of chaos*). Hence, for obtaining a lower log(MASE) it is necessary that $0.5\leq C_{dist}\leq 1$, S≥0.5, and E≤0.5. For $H_{spct}$ a similar relationship is observed in the sense that high *C* values are associated with lower log(MASE) due to E≤0.5 and S≥0.5 proportions. Notice that this *C* separates ARIMA performance into two performance regions, with the worst log(MASE) corresponding to complexities below 0.6, dropping even to C∼0 as the logistic map becomes more chaotic. In contrast, *C* for $H_{perm}$ and $H_{2reg}$ have larger complexity values for worse forecasting performance; $C_{2reg}$ separates ARIMA performance into two performance regions similar to $C_{spct}$.

#### 4.1.2. The CFS of All Synthetic Data

**Figure 4:**
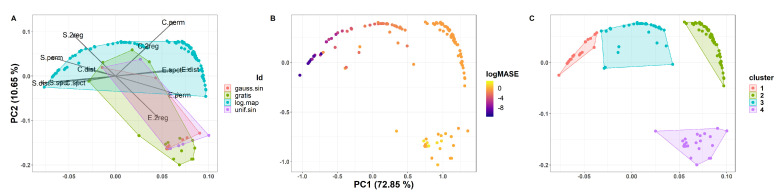
The Logistic Map and its ESC. The top plot shows the bifurcation diagram, whereas below the corresponding ESC for different entropy measures its showed. (**A**) ESC variables are projected into the Complexity Feature Space (CFS) plane to display its loadings; (**B**) Two dimension Time Series are colored in accordance to its log(MASE); (**C**) K-means clustering algorithm results using four centroids.

All the synthetic data were mapped as 2D point in the CFS which is displayed in Figure 4.

In Figure 4A ESC variables are projected into the CFS plane to display its loadings. Notice that the first two Principal Components (PCs) explain a large amount of the variance in the data (PC1∼73%,PC2∼10.6%), due to most of the series in the data set belonging to the logistic map. $C_{perm}$, $E_{dist}$, $E_{perm}$, and $E_{2reg}$ have positive loadings on the PC1, whereas $S_{dist}$, $S_{perm}$, and $S_{2reg}$ have negative loadings. $E_{spct}$ and $S_{spct}$ are parallel to its $H_{dist}$ counterpart, but with lower loadings on the PC1. The rest of the complexities have lower loadings in these two PCs. Convex hulls are used to denote each TS source; however, note that these are constrained to specific regions in the CFS. Hulls corresponding to the corrupted sine waves mostly overlap each other, and share a large portion with GRATIS data.

In Figure 4B 2D TS are colored in accordance to its log(MASE). Observe that a clockwise relationship between forecasting performance is displayed: ARIMA best performance lies in the upper left quadrant and its worst results on the lower right. It is interesting that the worst log(MASE) correspond to noisy time series, instead of the chaotic source, and that they are *conveniently* confined to specific regions in the CFS. By *convenient* we meant that a clustering algorithm may be used to cluster TS characterized by the ESC variables to obtain performance clusters, employed to determine if a forecasting method is useful or not for a given TS. Encouraged by the latter, the results, obtained by the popular K-means clustering algorithm using four centroids, are shown in Figure 4C. Notice that the resulting clusters correspond to the performance regions mentioned before.

### 4.2. Complexities and Forecastability of the M4 Competition TS

**Figure 5:**
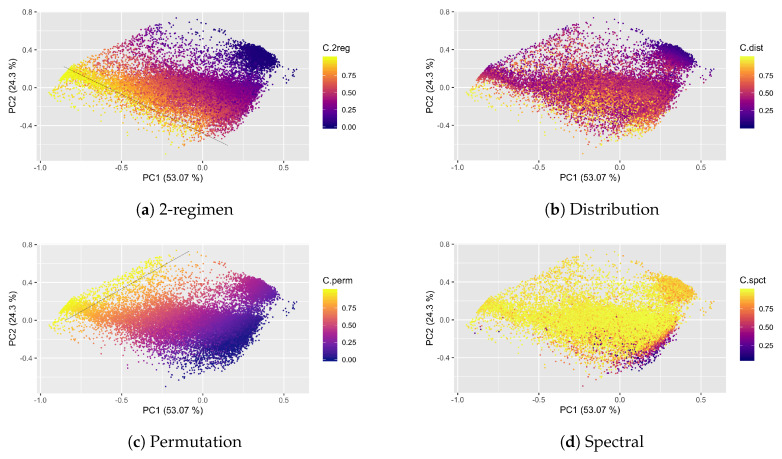
Four complexity measures and the Principal Components Analysis (PCA) of 12 features (ESC).

Before we delved into the analysis of M4 Competition results, we display the relationships of different ESC measures of the M4 set in the CFS. In Figure 5 all TS (Yearly, Quarterly, Monthly, Weekly and Daily) are displayed as 2-D points; we focus on the *C* measure for each entropy measure (Figure 5a 2-regimen, Figure 5b distribution, Figure 5c permutation, and Figure 5d spectral); colors range from brighter (corresponding to higher values C∼1) to darker (corresponding to lower values C∼0). Observe that both $C_{2reg}$ and $C_{perm}$ achieves the line gradient behavior with high complexity values as the PC1 becomes more negative, and lower complexity values as it becomes more positive. Interestingly, regarding PC2, they are on opposite sides. On the other side, $C_{dist}$ visual gradient is perceived more on the y-axis (lower values are positive and higher values are negative), in contrast to the PC1 where no clear relationship between high and low *C* values is observed. Similarly, $C_{spct}$ shows high values over most of the two PCs plane. However, for both $C_{dist}$ and $C_{spct}$ this behavior can be product of the reduction of dimensionality by the linear method.

These intuitions are corroborated by the loadings of these variables on the four most important PCs, which are presented in Table 3. Notice that the PC1 and PC2 are mainly represented by Permutation and 2-regimen complexities. On the other hand, for PC3 the most significant variable is $C_{dist}$ which has a negative loading, whereas for PC4, the most significant variable is $C_{spct}$. In particular, the *C* part of the ESC measures will be used for the analysis in Section 4.3. Table 4 shows results for the explained variance proportion corresponding to each principal component. Observe that the first two PCs account for most of the variance (≈77%) in data, and with only 4 PCs we account for the 100% of the variance.

**Figure 6:**
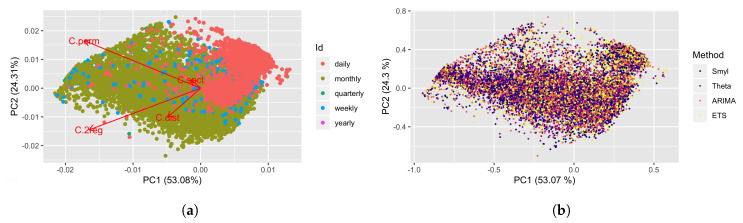
Analysis of TS regarding its Period frame andWinning method by TS. (**a**) Selected M4 Time Series are shown in the Complexity Feature Space (CFS), and each one is colored according to the period of its frequency; (**b**) M4 Time Series are colored according to the winning method.

In Figure 6a selected M4 TS are shown in the CFS color-coded by the period that corresponds to its frequency. Observe that Daily and Monthly TS are readily identifiable in the 2d projection, while the former is restrained to a specific region of the CFS, and the latter is spread across the CFS. Weekly TS are constrained to the middle section of the CFS, while Yearly and Quarterly TS are barely noticeable. On the other hand, in Figure 6b M4 TS are shown colored in accordance to the winning method, where 6838 points correspond to ARIMA, 6384 correspond to the SMYL, 5064 to the ETS, and 4324 to the Theta. It is worth mentioning that even when ARIMA wins in more TS than the Smyl algorithm, error magnitudes of the former are larger in comparison to the latter. Moreover, there are no specific regions in which any of the tested methods obtain better performance than the rest, which is consistent with the *No-Free Lunch* theorem.

**Figure 7:**
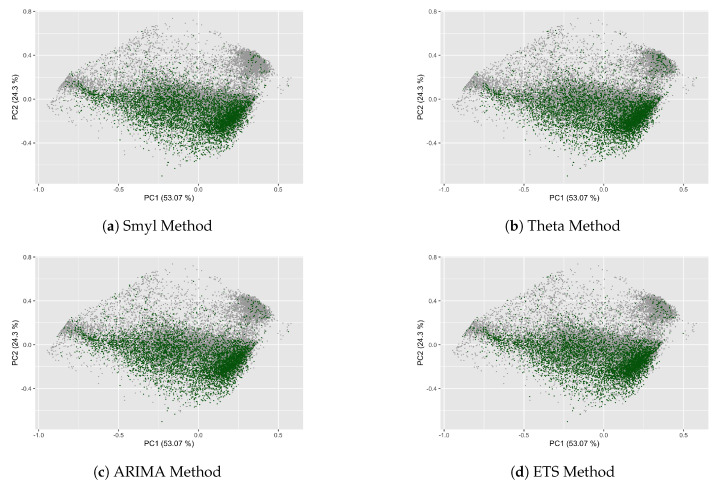
Relationship between *log(MASE)* and Complexity measures of the first quartile.

Continuing with the experiments on M4 dataset, one of our main interests is to determine the forecastability of the M4 Competition through the complexity measures of TS. Therefore, we consider four methods of M4-Competitions in order to establish whether there exists or not a relationship between the MASE error (log(MASE) to effects of functionality) by forecasting method (Smyl, Theta, ARIMA, and ETS). The first activity was to divide the complete dataset into four quartiles, in Figure 7, with each gray point representing one TS that belongs to the complete dataset of M4 Competition, and the dark green point representing the TS whose (log(MASE) value is found of the first quartile; specifically, in Figure 7a, the (log(MASE) values corresponding to the Smyl forecasting method, the Figure 7b, the (log(MASE) values corresponding to the Theta forecasting method, and so on, this figure shows that the TS with low log(MASE) value are concentrated in the negatives values of the second principal component and has a high value for the first principal component, according to Figure 6a; this kind belongs mainly to the Monthly period.

**Figure 8:**
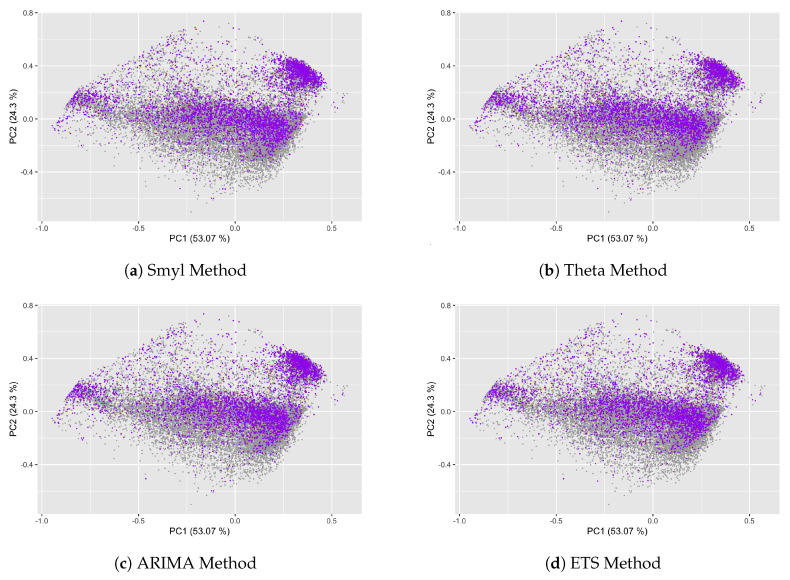
Relationship between *log(MASE)* and Complexity measures of the fourth quartile.

In the same way, Figure 8 represents the TS that integrates the four quartiles according to the log(MASE) values, where the purple point corresponds to the TS that belongs to this quartile. Making a comparison between Figure 8a–d it is noted that the log(MASE) values for each one of forecast methods is closer between them, and in terms of distribution area for these TS, we determine that when the complexity measures are higher, the log(MASE) value is higher too; moreover, compared to the distribution of TS by periods (Yearly, Quarterly, Monthly, Weekly and Daily), the major part of Daily TS belongs to this quartile.

### 4.3. Regression Results

After analyzing the TS applying Principal Component Analysis, we used the Principal Components Regression method (PCR) to adjust a model of linear regression by least squares using the four components generated on a previous subsection. For this test, we select the TS by period and divide them in a subset of training and another subset of test with 80% and 20%, respectively. The estimated error of prediction was calculated with the Mean Square Error (MSE), and the results are presented in Table 5, the best prediction values were obtained in the Quarterly and Weekly periods, and the high prediction error was obtained in the Yearly period; it is important to remember that the subset of Yearly period is composed only of 56 TS.

## 5. Conclusions

In this work, we proposed four possible characterizations of the state of a dynamic system based on Shannon entropy: a frequentist binning approach (distribution), the spectral probability density of the TS (spectral), and symbolic transformations (permutation and 2-regimes) defining the alphabet by ordinal rank patterns, and sequences of the first derivative sign. These characterizations are the measures of complexity, and they are bounded between zero (i.e., minimal Entropy/Complexity) and one (i.e., maximal Entropy/Complexity). One important feature for these measures is that Entropy is maximal when TS states are equiprobable. In contrast, Complexity is maximal when the system tends to high Self-Organization or high Emergence (i.e., discernible patterns with some noise or high noise with some discernible patterns). From those measures, we determined the principal components, and through its loadings, we found that $C_{perm}$ and $C_{2reg}$ are those measures that represent patterns that identify TS groups with similar features. Also, by plotting the TS by its log(MASE) in different quartiles, we observed that the TS with low log(MASE) are concentrating along with the first principal component. Moreover, comparing the four forecasting methods, the behavior is very similar between them; it is important to emphasize that for every TS, the log(MASE) values displayed in this space are very close among each other. Thus, these plots only corroborate the supposition that the winning method is the best for the quantity of TS where the winning is individually. Another important result is that we found that from the four forecasting methods identified as the winner of each TS dispersed over the complete TS, we see that the two principal components are consistent with the *No-free lunch* theorem. Finally, we determine that the TS with complexity measures closer to zero correspond to a low log(MASE) error, whereas when complexities measures are high, the log(MASE) tends to be high.

## Figures and Tables

**Table 1:** M4 Competition time series.

								Selected Series	
Frequency	Demographic	Finance	Industry	Macro	Micro	Other	Total	Size	%
Yearly	1088	6519	3716	3903	6538	1236	23,000	56	0.24%
Quarterly	1858	5305	4637	5315	6020	865	24,000	256	1.07%
Monthly	5728	10,987	10,017	10,016	10,975	277	48,000	18,406	38.35%
Weekly	24	164	6	41	112	12	359	293	81.62%
Daily	10	1559	422	127	1476	633	4227	3599	85.14%
Hourly	0	0	0	0	0	414	414	0	0.00%
Total	**8708**	**24,534**	**18,798**	**19,402**	**25,121**	**3437**	**100,000**	**22,610**	**22.61%**

**Table 2:** Example of a converted time series for codifying TS values.

49	52	53	61	71	67	72	52	48	…	54
–	1	1	1	1	0	1	0	0	…	1

**Table 3:** Principal Components Analysis (PCA) results.

	PC1	PC2	PC3	PC4
C.2reg	−0.6768	−0.5947	0.4336	0.0142
C.dist	−0.2003	−0.4150	−0.8776	−0.1323
C.perm	−0.7057	0.6777	−0.1757	0.1086
C.spct	−0.0607	0.1219	0.1047	−0.9851

**Table 4:** Proportion of variance for the principal components.

	PC1	PC2	PC3	PC4
Standard deviation	0.2923	0.1978	0.1592	0.1052
Proportion of Variance	0.5308	0.2431	0.1574	0.0687
Cumulative Proportion	0.5308	0.7739	0.9313	1.0000

**Table 5:** Mean Square Error of prediction with a linear regression model.

	Yearly	Quarterly	Monthly	Weekly	Daily
MSE	115.0187	6.8431	21.1561	4.3047	56.2699
